# Downtrends in Offside Offenses Among ‘The Big Five’ European Football Leagues

**DOI:** 10.3389/fpsyg.2021.719270

**Published:** 2021-09-20

**Authors:** Yangqing Zhao

**Affiliations:** School of Physical Education and Health, Wenzhou University, Wenzhou, China

**Keywords:** soccer, football, offside offenses, European big five leagues, VAR

## Abstract

This study examined the evolution of offside offenses and pass performance across a 10-season period in the top five European soccer leagues. Match performance observations (*n* = 18 259) were analysed for emergent trends. Two-way ANOVA analyses revealed significant league and seasonal differences among the five leagues (medium effect size). The total offside offenses committed during a match experienced a clear decline during the 10 seasons. In contrast, moderate increases were evident for all passing differential variables. Offside offenses per match were higher in the German Bundesliga and Spanish La Liga than in the English Premier League and France Ligue 1. However, the English Premier League had the greatest value in the touch differential, pass differential, successful pass differential, and key pass differential among all leagues. It is important to note that the number of offside offenses fell after the implementation of VAR.

## Introduction

The offside rule is a unique feature of soccer matches. It contributes significantly to the dynamism of the game and adds sophistication and creativity to soccer. However, the offside rule is also the source of a large amount of goal-related controversy and has experienced long-term changes (Table 1). The offside rule became increasingly liberalised until it was finally amended in 2005. According to FIFA Law 11, a player is in an offside position “if he is nearer to the opponents’(defenders) goal line than both the ball and the second-last opponent (defender). A player in an offside position is only penalised if, at the moment the ball is played by one of his team, he is, in the opinion of the referee, involved in active play by interfering with play, or interfering with an opponent, or gaining advantage being in that position”.

**TABLE 1:** Brief history of the offside rule.

Year	Law change
1863	A player is offside if he is in front of the ball.
1866	Forward passes are made legal, provided that there are 3 defenders between the receiver and the goal. Previously, all attacking players in front of the ball were offside.
1907	A player receiving a pass cannot be offside if he is on his own half.
1920	Impossible to be offside after a throw-in.
1925	The Offside Rule (see 1866) is reduced from three to two defending players.
1990	No longer offside if the receiving player is even with 2nd-to-last defender.
2003	To be offside, a player has to either touch the ball or be in a position to potentially make physical contact with an opponent.
2005	In the definition of an offside position, “nearer to his opponents’ goal line” means that any part of his head, body or feet is nearer to his opponents’ goal-line than both the ball and the second-to-last opponent. The arms are not included in this definition.

The phrase “nearer to his opponents’ goal line” refers to those body parts that could be used to score a goal (i.e., all body parts except arms and hands). It follows from the rule that judging offside includes two subtasks. The first subtask requires perceiving the position of the forward from team A in relation to the last defender of team B (who is, in most cases, the second last opponent before the goal keeper) at the moment when another player from team A touches or plays the ball. The second subtask requires judging whether the forward is actively involved in play or gaining an advantage from being in the offside position (Wühr et al., 2015).

The literature about offside derives from two main approaches: offside judgment and comparative analysis on offside.

First, greater attention has been paid in the literature to the examination of offside decision-making process in assistant referees and errors associated with it. Baldo et al. (2002) were the first to propose the flash-lag effect as a possible source of incorrect offside judgements. Helsen et al. (2006) and Gilis et al. (2008) examined its validity to explain errors made by assistant referees. Catteeuw et al. (2010) indicated that flag errors can best be explained by the perceptual illusion induced by the flash-lag effect. On the other hand, in line with the optical error hypothesis (Oudejans et al., 2000, 2005) that may explain why incorrect offside decisions may occur, Helsen et al. (2006) revealed that 26.2% of the offside situations were assessed incorrectly during the 2002 World Cup in Japan and Korea, most of which were flag errors (when the assistant referee gave an offside by raising his flag, while the attacker was in an onside position). Furthermore, Oudejans et al. (2007) agreed that scoring incorrect flag decisions is very objective and clear. However, they also suggested examining the non-flag situations in more detail. In this respect, Oudejans et al. (2005, 2007) defined an offside situation as follows: “an offside situation is a situation in which the ball was passed toward the opponents’ goal line and in the direction of a receiving attacker who was positioned within a few metres of the offside line”.

As regards sportsmen, in soccer (association football), the ability to avoid offside situations has been appointed as a factor that distinguishes players’ positional roles, match outcomes, and game location. In terms of players’ positional roles, forward commit more offside offenses than fullbacks (Taylor et al., 2004), and winning teams commit significantly more offside offenses than losing teams do (Lago-Peñas et al., 2010; Zhou et al., 2018). Moreover, studies show that home teams commit significantly higher numbers of offside offenses than visiting teams do (Lago-Peñas and Lago-Ballesteros, 2011). However, significant differences across sections of the league table were not found for offside offenses in the Spanish La Liga 2008–2009 season (Lago-Ballesteros and Lago-Peñas, 2010). The offside offenses were similar for winning, drawing and losing teams in 288 matches played at the group stage in the UEFA Champions League in the 2007-2008, 2008-2009, and 2009–2010 seasons (Lago-Peñas et al., 2011). Furthermore, offside offenses during the game have been shown to not be determinant in achieving offensive success (Schauberger et al., 2017).

The most important limitation of previous research concerns the issue of general developments in offside offenses. Scholars have long focused on judgement or flag error with offside offenses rather than longitudinal analysis (Gilis et al., 2009; Catteeuw et al., 2010; Huttermann et al., 2017), which focuses on the evolution of the offside rule. To our knowledge, gaps in the passing and development of offside offenses between the top European leagues during the last decade have not been explored. Accordingly, the aim of this study is to examine the league and seasonal effects on the trends of offside offenses and passing difference.

This study will help us characterise the magnitude of dynamic change of offside offenses and passing difference. Taking into account the introduction of Video assistant referees (VAR) and the widening of the gap in strength between teams, we hypothesised that offside offenses would experience a significant decline. To test this hypothesis we collected offside data for 10 seasons in the top five European leagues and analyzed the seasonal and league effects. And we further try to explain the reason for the downward trend through the analysis of passing difference data.

## Materials and Methods

### Samples

Data from the first divisions in the English Premier League (EPL,3800 matches), French Ligue 1 (Ligue 1,3800 matches), German Bundesliga (Bundesliga,3060 matches), Italian Serie A (Serie A,3799 matches), and Spanish La Liga (Liga,3800 matches) were obtained through online sources (Whoscored.com) with permission. The data resources from Whoscored.com are supported by OPTA Sportsdata Company. The reliability of the tracking system (OPTA Client System) has been verified by Liu et al. (2013). They showed that the data collection system (OPTA Client System) achieved a sufficiently high inter-group consistency (Kappa coefficient between 0.86 and 0.94) when collecting real-time match data. Ethics committee approval of the current study was gained from the local university.

For comparisons between leagues, the most recent ten seasons were analysed, beginning with 2009/2010, when data on all leagues of interest were available. The number of offside offenses, touches, passes and successful passes for home and away teams were obtained for each individual match and were analysed per season for each league. Data for all variables were missing from one match in Serie A (Cagliari vs. Roma in 2012/2013 was forfeited), resulting in a total of 18,259 matches over 10 seasons, beginning with 2009/2010.

The match analysis included the coding of technical indicators based on the criteria defined by OPTA and included the number of offside offenses, touches (a sum of all events where a player touches the ball, which excludes things such as aerial lost or challenge lost), passes, successful passes, and key passes (the final pass from a teammate leading to a shot on goal). The difference between touches, passes, successful passes, and key passes is the absolute value derived by subtracting the respective value for the home team from that of the away team. Total offside offenses refers to the sum of offside offenses on both sides.

### Statistical Analysis

**FIGURE 1:**
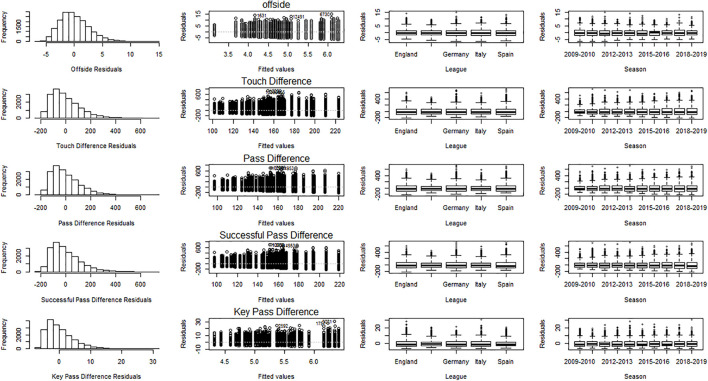
Model validation graphs. Histogram of the residuals (normality). Fitted values versus residuals (independence). Residuals versus league (homogeneity). Residuals versus season (homogeneity).

Longitudinal data were analysed using a two-way ANOVA with league and season as independent variables. Even minor deviations from normality can result in data with large sample sizes being classified as not normally distributed. We therefore prefer to assess normality, homogeneity, and independence purely based on a graphical inspection of the residuals (Figure 1).

In Figure 1, the distribution of the residuals appears skewed, which means the violation of normality. However, several authors argue that violation of normality is not a serious problem (Sokal and Rohlf, 1995, p407; Zar, 2010, p137) as a consequence of the central limit theory. Some authors even argue that the normality assumption is not needed at all provided the sample size is large enough (Fitzmaurice et al., 2012; Ghasemi and Zahediasl, 2012).

For every parameter presented in the present study, a significant interaction between these factors was identified (*p* < 0.05). Effect sizes were calculated using partial eta squared ($\eta_{p}^{2}$). The following scales were used to classify the effect size of the test (Cohen, 1992): very small, 0–0.02; small, 0.02–0.15; moderate, 0.15–0.35; large, 0.35–1.0. Tukey’s *post hoc* tests were used to compare leagues and seasons (Table 2). To control for type I error, Bonferroni’s correction was applied by dividing the α level by the number of pairwise comparisons being made. Thus, an operational α level of 0.005 (*p* < 0.05/10) was used for league comparisons, and an operational α level of 0.001 (*p* < 0.05/45) was used for season comparisons of each dependent variable.

**TABLE 2:** Offside offenses and passing performance across 10 seasons for European Big five leagues.

	League	Season									
		2009/2010	2010/2011	2011/2012	2012/2013	2013/2014	2014/2015	2015/2016	2016/2017	2017/2018	2018/2019
Offside offenses	EPL	4.6 ± 2.4	4.7 ± 2.6	4.5 ± 2.7	4.6 ± 2.4	4.2 ± 2.6	3.9 ± 2.2^ABD^	3.9 ± 2.2^ABD^	3.8 ± 2.3^ABCD^	4.1 ± 2.3	4.1 ± 2.3
	Ligue 1	4.3 ± 2.3^D^	4.4 ± 2.4^D^	4.6 ± 2.5^D^	5.6 ± 2.8	4.7 ± 2.4^D^	4.5 ± 2.2^D^	4.3 ± 2.5^D^	4.3 ± 2.3^D^	4.1 ± 2.3^ED^	3.1 ± 2.0^ABCDEFGHI^
	Bundesliga	6.3 ± 3.2	6.2 ± 3	6.1 ± 3.2	5.4 ± 2.7^AB^	4.8 ± 2.5^ABC^	4.9 ± 2.6^ABC^	5 ± 2.4^ABC^	4.6 ± 2.5^ABCD^	4.0 ± 2.4^ABCDEFG^	4.0 ± 2.5^ABCDEFG^
	Serie A	6.2 ± 3	5.9 ± 2.9	5.8 ± 2.8	5 ± 2.6^ABC^	4.2 ± 2.1^ABCD^	4.7 ± 2.7^ABC^	4.8 ± 2.4^ABC^	4.6 ± 2.3^ABC^	4.2 ± 2.4^ABCD^	3.7 ± 2.4^ABCDFGH^
	Liga	5.9 ± 2.8	5.8 ± 3.1	5.4 ± 2.6	5.2 ± 2.9^A^	4.8 ± 2.6^ABC^	4.9 ± 2.7^AB^	4.9 ± 2.7^AB^	4.7 ± 2.5^ABC^	5.1 ± 2.6^AB^	4.5 ± 2.6^ABCD^
Touch	EPL	123.2 ± 93.3	123 ± 92.4	149.8 ± 122.6	148.4 ± 113	170.3 ± 115.4**^AB^**	168.1 ± 122.8**^AB^**	152.5 ± 112.5**^AB^**	191.7 ± 127**^ABCDG^**	210 ± 167.9**^ABCDEFG^**	223.1 ± 153**^ABCDEFGH^**
	Ligue 1	101.6 ± 78.2	106.6 ± 82.8	128.2 ± 92.4	122.8 ± 89.4	136.3 ± 108.9**^AB^**	145.4 ± 110.3**^AB^**	166.2 ± 131.2**^ABCDE^**	166.5 ± 119.3**^ABCDE^**	168.2 ± 124.3**^ABCDE^**	157.5 ± 113.6**^ABCD^**
	Bundesliga	117 ± 92.9	119.6 ± 90.1	140.2 ± 110.3	148.1 ± 111.4	157.1 ± 141.7**^AB^**	177 ± 143.3**^ABC^**	196.3 ± 163.3**^ABCDE^**	198.9 ± 147.3**^ABCDE^**	168.5 ± 135.4**^AB^**	183.8 ± 149.9**^ABCD^**
	Serie A	107.3 ± 79.4	109.8 ± 80	129.2 ± 104.9	119.3 ± 83.4	133 ± 97.6	154.3 ± 108.7**^ABD^**	168.4 ± 118.6**^ABCDE^**	167.9 ± 125.4**^ABCDE^**	177.1 ± 131.5**^ABCDE^**	164.9 ± 121.8**^ ABCDE^**
	Liga	134.3 ± 115.8	153.8 ± 147.3	165 ± 139.4**^A^**	166.8 ± 140.4**^A^**	159.6 ± 123.6	154.7 ± 125.5	151 ± 115.3	166.2 ± 121.9**^A^**	169.5 ± 124**^A^**	163.6 ± 138.4**^A^**
Pass	EPL	116.7 ± 89	117.7 ± 88.6	145.4 ± 118.6**^A^**	142.9 ± 108.9	164.4 ± 111.2**^AB^**	166.1 ± 121**^AB^**	151.2 ± 112**^AB^**	185.1 ± 124.5**^ABCDGH^**	206.5 ± 166.7**^ABCDEFG^**	220.1 ± 152.2**^ABCDEFGH^**
	Ligue 1	96.7 ± 75.6	104.4 ± 81	127.7 ± 91.8**^A^**	123.9 ± 89	134.3 ± 106.4**^AB^**	141.8 ± 108.2**^AB^**	163.9 ± 128.5**^ABCDE^**	159.8 ± 117.5**^ABCD^**	163 ± 120**^ABCDE^**	153.2 ± 111.7**^AB^**
	Bundesliga	115 ± 90.8	115.9 ± 88.6	137.8 ± 108.6	144.9 ± 107.7	153.7 ± 143.3**^AB^**	177.1 ± 144.2**^ABCD^**	194.7 ± 163.1**^ABCDE^**	195.6 ± 145**^ABCDE^**	165.5 ± 131.7**^AB^**	177.8 ± 148**^ABCD^**
	Serie A	105.4 ± 77.1	109.8 ± 81.3	131.3 ± 107.4	115.6 ± 82.2	130.7 ± 96.9	149.3 ± 106.4**^AB^**	166.1 ± 118.7**^ABCDE^**	161.9 ± 121.1**^ABCDE^**	174.4 ± 130.1**^ABCDE^**	160.8 ± 120.1**^ABCDE^**
	Liga	130.5 ± 111.7	152 ± 145.1	163.6 ± 138.6**^A^**	162.9 ± 138.8**^A^**	155.6 ± 120.7	152.9 ± 124	148.5 ± 115	159.4 ± 117.5**^A^**	163.9 ± 119.7**^A^**	159.2 ± 135.5**^A^**
Successful pass	EPL	118 ± 90.4	118.2 ± 89.4	145.5 ± 120.7	144.2 ± 109.8	167 ± 112.5**^AB^**	166.2 ± 122.2**^AB^**	151.5 ± 111.6**^ABH^**	186.6 ± 124.9**^ABCDG^**	206.6 ± 166.1**^ABCDEFG^**	218.8 ± 151.5**^ABCDEFGH^**
	Ligue 1	97.6 ± 77	104.8 ± 81.2	126.8 ± 91.3	122.6 ± 89.1	132.9 ± 105.6	145 ± 108.7**^A^**	163.8 ± 129**^ABCDE^**	161.8 ± 118.6**^ABCDE^**	163.4 ± 120.8**^ABCDE^**	153.5 ± 111.9**^AD^**
	Bundesliga	114.1 ± 91.4	116 ± 89.2	138.1 ± 108.5	145.6 ± 107.6	152.8 ± 143.2**^AB^**	175.6 ± 143.5**^ABC^**	193.7 ± 163.9**^ABCDE^**	194.2 ± 143.8**^ABCDE^**	164.9 ± 132.8**^AB^**	178.1 ± 147.8**^ABCD^**
	Serie A	105.6 ± 78.8	110.1 ± 80.1	130.9 ± 105.7	118.1 ± 83.5	132.3 ± 97.5	150.8 ± 107**^ABD^**	167.8 ± 119.1**^ABCDE^**	163 ± 122.3**^ABCDE^**	175.1 ± 130.8**^ABCDE^**	161.9 ± 120.5**^ABCDE^**
	Liga	130.3 ± 111.9	151.7 ± 146**^A^**	165 ± 139.7**^A^**	164.1 ± 139.6	157.1 ± 122.3	154.2 ± 124.6	149.4 ± 114.7	159.3 ± 117.5**^A^**	163.9 ± 119.3**^A^**	158.1 ± 135.6
Key pass	EPL	6.2 ± 5.1	5.7 ± 4.4	6.3 ± 4.6	6.2 ± 4.5	6.2 ± 4.9	5.8 ± 4.3	5.4 ± 4.5	6.4 ± 4.9^G^	6.2 ± 4.7	5.8 ± 4.7
	Ligue 1	4.9 ± 3.8	4.7 ± 3.7	5.1 ± 4.1	4.9 ± 3.7	4.7 ± 3.7	4.3 ± 3.4	4.6 ± 3.6	4.9 ± 3.7	4.9 ± 3.9	4.9 ± 3.9
	Bundesliga	5.1 ± 4.2	4.9 ± 3.9	5.5 ± 3.8	5.1 ± 4.2	5.4 ± 4.3	5.5 ± 4.2	5.6 ± 4.6	5.1 ± 4.2	5.2 ± 4.1	5.6 ± 4.2
	Serie A	5.2 ± 4.1	4.8 ± 3.7	5.2 ± 4	5.4 ± 4.3	5.2 ± 4.3	5.1 ± 4	5.6 ± 4.5	5.9 ± 4.4^B^	6.3 ± 4.9^ABC^	6.3 ± 5^ABC^
	Liga	5.7 ± 4.5^G^	5.5 ± 4.3^G^	5.4 ± 4.4	5.3 ± 4	5.2 ± 4.3	5 ± 3.8	4.4 ± 3.7	5 ± 3.9	4.7 ± 3.8	4.7 ± 4

*Data are presented as means and standard deviations. A denotes the difference from the 2009/2010 season (adjusted *p* < 0.05); B denotes the difference from the 2010/2011 season (adjusted *p* < 0.05); C denotes the difference from the 2011/2012 season (adjusted *p* < 0.05); D denotes the difference from the 2012/2013 season (adjusted *p* < 0.05); E denotes the difference from the 2013/2014 season (adjusted *p* < 0.05); F denotes the difference from the 2014/2015 season (adjusted *p* < 0.05); G denotes the difference from the 2015/2016 season (adjusted *p* < 0.05); H denotes the difference from the 2016/2017 season (adjusted *p* < 0.05); I denotes the difference from the 2017/2018 season (adjusted *p* < 0.05).*

All analyses were conducted using SPSS Statistics for Windows, version 19.0, and data visualisation was carried out using the R statistical programming language and GraphPad Prism version 7.0.

## Results

### Offside Offenses

**FIGURE 2:**
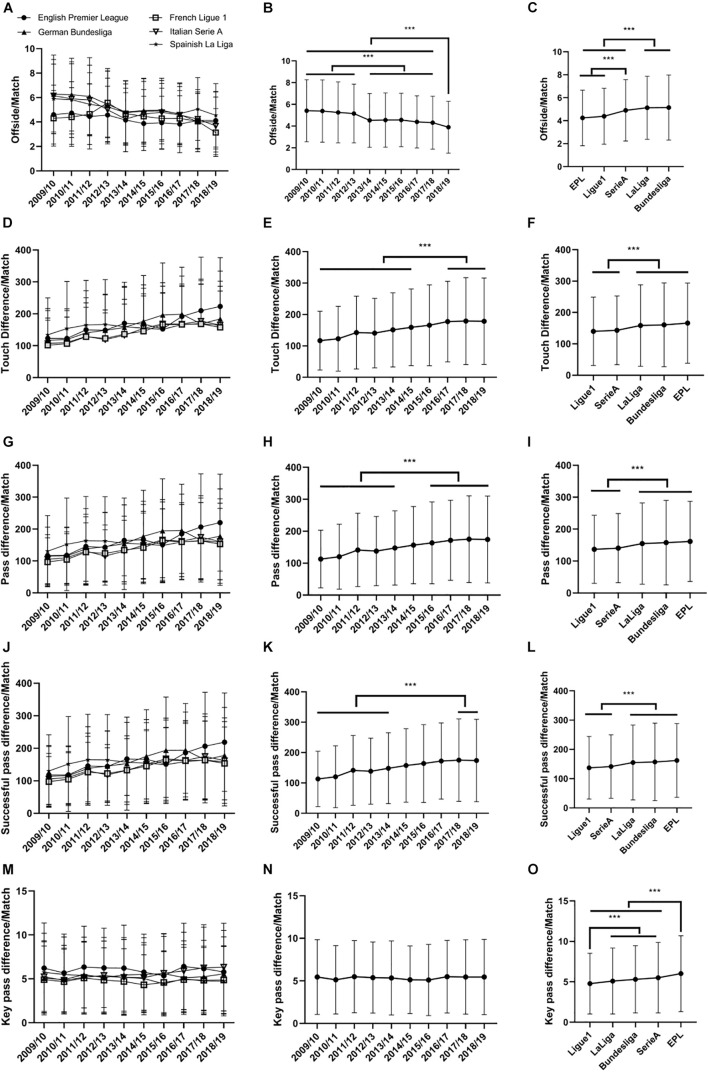
**(A)** Offsides per match, **(D)** touch difference per match, **(G)** pass difference per match, **(J)** successful pass difference per match, **(M)** and key pass difference per match for each league over the ten seasons span. **(B)** Offsides per match, **(E)** touch difference per match, **(H)** pass difference per match, **(K)** successful pass difference per match, **(N)** and key pass difference per match for each season. **(C)** Offsides per match, **(F)** touch difference per match, **(I)** pass difference per match, **(L)** successful pass difference per match, **(O)** and key pass difference per match for each league. Data are shown as mean ± SD. ***Denotes statistical significance at the 0.1% level.

Figure 2A shows the total number of offside offenses called per match for each league over the ten-season span. Two-way ANOVA revealed a significant effect of season [*F*(9,18209) = 78.74, $\eta_{p}^{2}=0.04$] and league [*F*(4,18209) = 99.42, $\eta_{p}^{2}=0.02$], with a significant interaction effect [*F*(36,18209) = 9.02, $\eta_{p}^{2}=0.02$] (all *p* < 0.0001). The number of offside offenses per match consistently declined in all leagues over the ten seasons, and there were significantly more total offside offenses called per match in the first four seasons compared with the last six seasons (all adjusted *p* < 0.0001, Figure 2B). From Figure 2C, it can be seen that there were significantly fewer offside offenses called in the EPL and League 1 compared to the other three leagues (adjusted *p* < 0.0001).

Simple main effects analysis demonstrated the clear seasonal effect in the EPL [*F*(9,18209) = 6.21, $\eta_{p}^{2}=0.003$], Ligue 1 [*F*(9,18209) = 21.20, $\eta_{p}^{2}=0.01$], Bundesliga [*F*(9,18209) = 33.55, $\eta_{p}^{2}=0.02$], Serie A [*F*(9,18209) = 38.21, $\eta_{p}^{2}=0.02$] and La Liga [*F*(9,18209) = 12.84, $\eta_{p}^{2}=0.01$] (all *p* < 0.0001). Pairwise comparison showed that there were significantly fewer offside offenses committed in 2018/2019 than in the other nine seasons for Ligue 1 (adjusted *p* < 0.0001) and significantly fewer offside offenses committed in 2018/2019 than in the first eight seasons for Serie A (adjusted *p* < 0.0001). La Liga committed fewer offside offenses in 2018/2019 compared with the first four seasons, but there were no differences between 2018/2019 and 2017/2018. For Bundesliga, there were significantly fewer mean offside offenses in the most recent two seasons than in the first seven seasons, but there were no differences between the recent two seasons and the other eight seasons for the EPL.

Furthermore, the EPL showed a smaller reduction than the other leagues. The number of offenses in the EPL decreased by 11% from the 2009/2010 season to the 2018/2019 season, while the decreases were 36% for the German Bundesliga, 40% for the Italian Serie A, 23% for the Spanish La Liga, and 27% for the French Ligue 1.

### Touches

Two-way ANOVA on touch differential per match revealed significant main effects of season [*F*(9,18209) = 66.00,$\eta_{p}^{2}=0.03$] and league [*F*(4,18209) = 34.15, $\eta_{p}^{2}=0.01$] as well as an interaction effect [*F*(36,18209) = 5.33, $\eta_{p}^{2}=0.01$] (all *p* < 0.0001). The touch differential per match consistently increased in all leagues over the ten seasons (Figure 2D). From Figure 2E, it can be seen that the touch differential per match was higher in the last three seasons (2016-2019) than in the first six seasons (all adjusted *p* < 0.0001).

Simple main effects analysis demonstrated the clear seasonal effect in the EPL [*F*(9,18209) = 30.48, $\eta_{p}^{2}=0.02$, *p* < 0.0001], Ligue 1 [*F*(9,18209) = 16.44, $\eta_{p}^{2}=0.01$, *p* < 0.0001], Bundesliga [*F*(9,18209) = 18.50, $\eta_{p}^{2}=0.01$, *p* < 0.0001], Serie A [*F*(9,18209) = 18.48, $\eta_{p}^{2}=0.01$, *p* < 0.0001] and La Liga [*F*(9,18209) = 2.93, $\eta_{p}^{2}=0.01$, *p* = 0.002]. Pairwise comparison showed that the touch differential in the most recent six seasons were significantly higher than in the first two seasons for all leagues except for La Liga (adjusted *p* < 0.05) and significantly higher in the most recent four seasons than in the first four seasons for the EPL, Serie A, and Ligue 1 (adjusted *p* < 0.05). La Liga had a higher touch differential in 2018/2019 than in 2009/2010, but there was no differential among the most recent nine seasons.

The EPL showed a larger increase than the other leagues (Figure 2F). In the EPL, the touch differential per match increased by 81% from the 2009/2010 to 2018/2019 season, while the increase was 57% for the German Bundesliga, 54% for the Italian Serie A, 22% for the Spanish La Liga, and 55% for the French Ligue 1.

### Passes

When leagues are compared by the pass differential per match (Figure 2I), Ligue 1 and Serie A are consistently lower than each of the other three leagues (adjusted *p* < 0.0001). The results of the ANOVA showed significant effects of season [*F*(9,18209) = 65.13, $\eta_{p}^{2}=0.03$], league [*F*(4,18209) = 32.25, $\eta_{p}^{2}=0.01$], and interaction [*F*(36,18209) = 5.78, $\eta_{p}^{2}=0.01$] (all *p* < 0.0001). The pass differential per match increased over time for all five leagues and in the ten seasons. In comparison, the most recent four seasons had higher pass differentials for each league than the first five seasons (Figure 2H).

Simple main effects analysis demonstrated the clear seasonal effect in the EPL [*F*(9,18209) = 32.58, $\eta_{p}^{2}=0.02$, *p* < 0.0001], Ligue 1 [*F*(9,18209) = 15.74, $\eta_{p}^{2}=0.01$, *p* < 0.0001], Bundesliga [*F*(9,18209) = 19.13, $\eta_{p}^{2}=0.01$, *p* < 0.0001], Serie A [*F*(9,18209) = 17.47, $\eta_{p}^{2}=0.01$, *p* < 0.0001] and La Liga [*F*(9,18209) = 2.77, $\eta_{p}^{2}=0.001$, *p* = 0.003].

Pairwise comparison showed that the pass differential was significantly higher in the recent five seasons than in the first two seasons for all leagues except for La Liga (adjusted *p* < 0.05) and a significantly higher pass differential in 2018/2019 than in the first eight seasons for the EPL (adjusted *p* < 0.05). La Liga had a higher pass differential in 2018/2019 than in 2009/2010, but there were no differences among the most recent nine seasons.

The EPL showed a larger increase than the other leagues (Figure 2G). The pass differential per match in the EPL increased by 89% from the 2009/2010 season to the 2018/2019 season, while the increase was 55% for the German Bundesliga, 53% for the Italian Serie A, 22% for the Spanish La Liga, and 58% for the French Ligue 1.

### Successful Passes

The results were similar, though not as consistent, for the successful pass differential per match Figure 2J. Analysis showed a significant effect of season [*F*(9,18209) = 64.68, $\eta_{p}^{2}=0.03$] and league [*F*(4,18209) = 31.03, $\eta_{p}^{2}=0.01$], along with a significant interaction effect [*F*(36,18209) = 5.64, $\eta_{p}^{2}=0.01$] (all *p* < 0.0001). Figure 2K shows an increase in the successful pass differential per match over the ten-season span. Ligue 1 and Serie A were lower than all other leagues (adjusted *p* < 0.0001).

Simple main effects analysis demonstrated a clear seasonal effect in the EPL [*F*(9,18209) = 31.58, $\eta_{p}^{2}=0.02$, *p* < 0.0001], Ligue 1 [*F*(9,18209) = 16.03, $\eta_{p}^{2}=0.01$, *p* < 0.0001], Bundesliga [*F*(9,18209) = 18.59, $\eta_{p}^{2}=0.01$, *p* < 0.0001], Serie A [*F*(9,18209) = 17.62, $\eta_{p}^{2}=0.01$, *p* < 0.0001] and La Liga [*F*(9,18209) = 2.87, $\eta_{p}^{2}=0.001$, *p* = 0.002]. Pairwise comparison showed that the successful pass differential was significantly higher in the most recent four seasons than in the first two seasons for all leagues except for La Liga (adjusted *p* < 0.05) and a significantly higher successful pass differential in 2018/2019 than in the first eight seasons for the EPL (adjusted *p* < 0.05). La Liga had a higher pass differential in 2017/2018 than in 2009/2010, but there were no differences among the most recent nine seasons.

The EPL showed a larger increase than the other leagues (Figure 2L). The successful pass differential per match in the EPL increased by 85% from the 2009/2010 season to the 2018/2019 season, while the increase was 56% for the German Bundesliga, 53% for the Italian Serie A, 21% for the Spanish La Liga, and 57% for the French Ligue 1.

### Key Passes

The analysis of key pass differential per match yielded a significant effect of league [*F*(4,18209) = 45.81, $\eta_{p}^{2}=0.01$, *p* < 0.0001] (Figure 2O). The EPL had a significantly higher differential than all other leagues (*p* < 0.0001). From Figures 2M,N, it can be seen that there is no significant difference in the key pass differential per match among the ten seasons.

Simple main effects analysis demonstrated the seasonal effect in the EPL [*F*(9,18209) = 2.59, $\eta_{p}^{2}=0.001$, *p* = 0.01], Serie A [*F*(9,18209) = 5.53, $\eta_{p}^{2}=0.003$, *p* < 0.0001] and La Liga [*F*(9,18209) = 3.34, $\eta_{p}^{2}=0.002$, *p* < 0.0001]. Pairwise comparison analysis showed that the key pass differential was significantly higher in the most recent two seasons than in the first three seasons for Serie A (adjusted p < 0.05) and significantly lower in 2015/2016 than in 2016/2017 for the EPL (adjusted *p* < 0.05). La Liga had a lower key pass differential in 2015/2016 than in 2009/2010 and 2010/2011, but there were no differences among the most recent eight seasons. However, there were no differences among all seasons in Bundesliga and Ligue 1.

## Discussion

The present longitudinal study is the first to map the evolution of offside offenses and pass differential parameters related to the top five European soccer leagues across 10 seasons. It was envisaged that the present study would improve our understanding of evolving patterns of offside offenses and various pass differential parameters during the last 10 seasons. Our data show that the total offside offenses per match declined monotonically in the most recent ten seasons, while three pass differential variables (touch, pass, and successful pass) continued to expand during the same time- period.

Offside offenses are at an historic low and falling. However, now that the game has changed, so has the law. Football has changed faster in the last 10 years than anyone has realised. The old end-to-end game of turning teams, getting in behind players, and trying to catch them offside, is dying at the top level. In modern football, the strategy does not tend to come from a striker gambling from behind the centre-backs. Top-level soccer in Europe is more organised and more technical, with less of the risky ambition that causes offside offenses. For instance, using a high-pressure style of play against a team that utilises a possession style of play could be very effective for regaining the ball and increasing the chances of scoring opportunities (Fernandez-Navarro et al., 2016). And successful teams from European Leagues and World Cups tend to have higher attacking third regains (Garganta et al., 1997; Bell-Walker et al., 2006). And ‘maintain possession’ strategy may involve more slow play with defensive movements, less risk when passing, and greater emphasis on re-gaining possession relative to teams who might place less importance on this strategy (Jones et al., 2004; Wright et al., 2011). Guardiola’ Barcelona, which relied on a sophisticated combination of possession and pressing that, in turn, leaded to the most fruitful period, both in reputation and in the number of titles achieved, including 14 titles during four seasons (Buldu et al., 2018).

As teams change how they attack, they also change how they defend. Modern soccer strategies and tactics are more focused on defensive aspects (Bangsbo and Peitersen, 2002). The 2005 rule change redefined what it means to be “interfering with play” in an offside position, namely, that a player either has to touch the ball or have the “potential for physical contact” with a defender. The rule created a major shift of activity in offside positions. Players could freely run offside and not receive the ball, only to be legally passed the ball by an onside team mate in the next phase. Suddenly, the offside line was no guarantee for defence any more. Attackers could break the line and still hurt their opponents. Therefore, defenders had to think differently. If a defender can now be hurt by attackers from behind, it might be safe to keep attackers in front instead. Defenders have become more flexible and more willing to drop deep. Such style of defending is characterised by a team collectively maintaining a compact shape in a zone nearer to their goal, and only applying pressure on their opponents when the attacking play begins to reach this zone (Bangsbo and Peitersen, 2002).

When compared to successful teams, the most effective scoring pattern for unsuccessful teams is set-play goals (Zhao and Zhang, 2019). Due to the sanctioning of offside positions after a corner kick or throw-in, an unsuccessful team can score without risking an offside call.

Video assistant referee were fully adopted by Bundesliga, Serie A, La Liga, Ligue 1, and the EPL in 2017/2018, 2017/2018, 2018/2019, 2018/2019, and 2019/2020, respectively. Our study suggests that in the three-season interval (2016/2017–2018/2019), there was a decrease in the number of offside calls after the implementation of the VAR in Bundesliga, Serie A, La Liga, and Ligue 1. The VAR effect was significant in Ligue 1 (the number of offside offenses committed per match in 2018/2019 was significantly less than that in 2016/2017 and 2017/2018) and Italy (the number of offside offenses committed in 2018/2019 was significantly less than that in the 2016/2017 season). However, due to not implementing the VAR, the EPL experienced a steady rise in offside calls. Thus, the introduction of VAR technology is one of the reasons for the recent decline of offside calls, and this finding is supported by previous research (Carlos et al., 2019).

Another interesting finding is that in the pre-VAR period, the five major European leagues (except for Ligue 1) also experienced a significant decline in offside offenses. The data demonstrate that all leagues (except for Ligue 1) committed fewer offside offenses during the recent three seasons (2014/2015-2016/2017) compared to the first two seasons (2009/2010–2011/2012).

For all leagues, the most pronounced increases in pass differential performance were for touches, passes and successful passes. Between the 2009/2010 and 2018/2019 seasons, a relative increase in the touch differential was observed for the EPL (45%), followed by Bundesliga, Serie A, Ligue 1, and La Liga (36, 35, 35 and 23%, respectively). A relative increase in the pass differential was observed for the EPL (47%), followed by Ligue 1, Bundesliga, Serie A, and La Liga (37, 35, 34 and 18%, respectively). Similar trends were also observed for successful pass differentials when year-on-year changes were calculated, discounting that a relative increase was observed for the EPL (46%), followed by Bundesliga, Ligue 1, Serie A, and La Liga (36, 36, 35 and 18%, respectively). Thus, it is reasonable to conclude that the gaps in touches, passes and successful passes have evolved for all of the top five leagues, albeit at different rates. Our results are in line with a previous study that reported that the highest ranked teams seem to adopt a more possession-based playing style than the bottom teams in the EPL, who still play a more direct style (Jones et al., 2004; Bloomfield et al., 2005; Taylor et al., 2008; Bradley et al., 2013).

Keeping hold of the ball, completing plays at a higher rate, and not surrendering the ball too often to the opposition means fewer offside offenses. Teams that have a greater share of passes force their oppositions to return to the backcourt, thus reducing offside offenses. The increase in the various pass differential indexes (touch, pass, successful pass) combined with the offside offense reduction illustrates the fact that the disparity in domination of the ball in the five major European leagues has increased significantly during the most recent ten seasons.

Our data demonstrate that whilst the touch, pass, successful pass differentials increased by ∼18–46% between 2009/2010 and 2018/2019, the key pass differential during matches remained relatively constant. Due to the lower key pass and pass conversion rate, we suggest that highly skilled teams are not better at passing than weaker teams. They simply engineer more easy passes in better locations and therefore limit their turnover. For example, the team will use backward passes to secure or support ball possession by passing the ball to a less advanced teammate to create space and new opportunities to attack (Fernandez-Navarro et al., 2016). Logically, the number of passes a team manages to complete in a match and a team’s passing quality do not have to go hand in hand.

A global measure of offside evolution of the different leagues noted that Bundesliga and La Liga committed more offside offenses than the EPL and Ligue 1.

Bundesliga had the greatest number of counterattacks in open-play situations than the EPL, La Liga, and Serie A (Mitrotasios et al., 2019). The running distance during the game (Schauberger et al., 2017) and the transitions between the attack and the defence (Vogelbein et al., 2014) have been shown to be the most important premise for a successful match. Thus, the high rhythm and speed of play in Bundesliga lead to more offside offenses committed.

La Liga teams, characterised by ball possession and technical players, favour the aesthetic side of the game and having greater control throughout the game (Sarmento et al., 2013; Castellano and Pic, 2019). The present findings demonstrate that there was no significant difference among the most recent nine seasons of La Liga in the touch, pass, and successful pass differentials. In contrast, due to the low possession rate for underdogs (Gollan et al., 2018), the EPL had significantly higher values of the above three indicators in the 2018/2019 season than in the first eight seasons.

This observation is mirrored by the fact that, over the period of the study, the disparity in ball dominance is more stable in La Liga, but the disparity in the Premier League has increased significantly. Pass volume is related to how often the ball is turned over. Those teams that complete passes at a higher rate are less prone to giving the ball back to the opposition. As no ball means no offside offense, this could be reflective of a reduction in offside offenses and therefore one of the reasons why the La Liga offside value is significantly higher than that of the EPL.

Concerning the limitations of the current study, three aspects should be highlighted. Contextual variables (e.g., opposition level and the score-line) were not taken into consideration and these variables may affect teams’ offside strategy. More matches, seasons, variables, and different competitions should be considered to provide conclusive descriptions and measures for playing styles and generalisability of the data. Finally, the combined effects of offensive and defensive tactics and opponent interactions should be included in the future. Thus, one interesting aim for future research is to include information on opposition level and the score-line. This information could further prove meaningful in explaining the decline of offside. And more samples could provide insights into the dynamic development of offside and pass difference.

More variables and matches should be considered to supply conclusive definitions for playing styles and generalisability of the data. Further research should attempt to establish the efficiency and effectiveness of playing styles when measuring performance and outcomes (i.e., scoring probability).

In summary, being offside is not a failure; it is just the price paid for gambles that do not pay out. Top-level soccer has become very tactical and defensive: many unsuccessful teams stopped playing the offside trap and began defending deeper and closer to the penalty box. Teams are not or cannot truly be looking to play an offside trap game as they did in the 1990s. Offside never dies, it just fades away.

## Conclusion

The most obvious finding to emerge from this study is that there has been a marked decline in the number of offside offenses committed during a match. By contrast, the touch, pass, successful pass differentials increased during the 10 seasons. Offside offenses per match were higher in the German Bundesliga tend to have greater value than the English Premier League and France Ligue 1. And the English Premier League had the greatest value in the touch differential, pass differential, successful pass differential, and key pass differential among all leagues. Furthermore, the number of offside offenses fell after the implementation of VAR.

## Data Availability Statement

The original contributions presented in the study are included in the article/supplementary material, further inquiries can be directed to the corresponding author/s.

## Author Contributions

The author confirms being the sole contributor of this work and has approved it for publication.

## Conflict of Interest

The author declares that the research was conducted in the absence of any commercial or financial relationships that could be construed as a potential conflict of interest.

## Publisher’s Note

All claims expressed in this article are solely those of the authors and do not necessarily represent those of their affiliated organizations, or those of the publisher, the editors and the reviewers. Any product that may be evaluated in this article, or claim that may be made by its manufacturer, is not guaranteed or endorsed by the publisher.
